# *Pv*D_1_ defensin, a plant antimicrobial peptide with inhibitory activity against *Leishmania amazonensis*

**DOI:** 10.1042/BSR20150060

**Published:** 2015-09-10

**Authors:** Viviane V. do Nascimento, Érica de O. Mello, Laís P. Carvalho, Edésio J.T. de Melo, André de O. Carvalho, Katia V.S. Fernandes, Valdirene M. Gomes

**Affiliations:** * Laboratório de Fisiologia e Bioquímica de Microrganismos, Centro de Biociências e Biotecnologia, Universidade Estadual do Norte Fluminense, Campos dos Goytacazes, Rio de Janeiro 28013-602, Brazil; † Laboratório de Biologia Celular e Tecidual, Centro de Biociências e Biotecnologia, Universidade Estadual do Norte Fluminense, Campos dos Goytacazes, Rio de Janeiro 28013-602, Brazil; ‡ Laboratório de Química e Função de Proteínas e Peptídeos, Centro de Biociências e Biotecnologia, Universidade Estadual do Norte Fluminense, Campos dos Goytacazes, Rio de Janeiro 28013-602, Brazil

**Keywords:** antimicrobial activity, *Leishmania amazonensis*, *Phaseolus vulgaris*, plant antimicrobial peptides, plant defensin

## Abstract

Plant defensins are small cysteine-rich peptides and exhibit antimicrobial activity against a variety of both plant and human pathogens. Despite the broad inhibitory activity that plant defensins exhibit against different micro-organisms, little is known about their activity against protozoa. In a previous study, we isolated a plant defensin named *Pv*D_1_ from *Phaseolus vulgaris* (cv. Pérola) seeds, which was seen to be deleterious against different yeast cells and filamentous fungi. It exerted its effects by causing an increase in the endogenous production of ROS (reactive oxygen species) and NO (nitric oxide), plasma membrane permeabilization and the inhibition of medium acidification. In the present study, we investigated whether *Pv*D_1_ could act against the protozoan *Leishmania amazonensis*. Our results show that, besides inhibiting the proliferation of *L. amazonensis* promastigotes, the *Pv*D_1_ defensin was able to cause cytoplasmic fragmentation, formation of multiple cytoplasmic vacuoles and membrane permeabilization in the cells of this organism. Furthermore, we show, for the first time, that *Pv*D_1_ defensin was located within the *L. amazonensis* cells, suggesting the existence of a possible intracellular target.

## Abstract (precis)

*Pv*D_1_ was able to inhibit the proliferation of *Leishmania amazonensis* promastigotes; *Pv*D_1_ caused cell membrane permeabilization and alterations in the cytoplasmic contents of these cells; *Pv*D_1_ was internalized in these cells, what suggests a possible intracellular target.

## INTRODUCTION

Leishmaniasis is an infectious disease that is prevalent worldwide, particularly in tropical and subtropical regions, killing thousands and debilitating millions of people each year. With 2 million new cases reported annually, infection by the *Leishmania* parasite represents an important global health problem for which there is no vaccine and few effective drugs [1].

The selection of an increasing number of antibiotic-resistant micro-organisms and other antimicrobial agents has attracted the attention of many researchers in an attempt to develop new therapeutic agents [2]. The therapeutic potential of AMPs (antimicrobial peptides) is enhanced owing to the ability of these compounds to rapidly kill a large number of micro-organisms such as bacteria, viruses, fungi and parasites that are multidrug-resistant [3,4]. There is an increasing interest in the study of AMPs because of their capacity to interact with certain cellular membranes, and the resulting antimicrobial activity that they display against pathogens [5].

Promising AMPs include the plant-derived defensins, a family of basic peptides which consists of 45–54 amino acids, arranged in three-dimensional structures formed by three antiparallel β-strands and one α-helix. This structure is stabilized by four disulfide bonds, which form a cysteine-stabilized α-helix β-strands motif, commonly found in these peptides [5,6]. The antimicrobial activity of plant defensins is mainly observed against fungi. However, some bacteria, particularly Gram-positive species, are also inhibited, although the activity is less pronounced than that against fungi. The growth of several fungal species, including several filamentous fungi and yeast cells, was inhibited when incubated with these peptides [7–21]. Despite the broad inhibitory activity that plant defensins exhibit against different micro-organisms, scientists know little about their activity against protozoa.

In a previous study, we isolated a plant defensin named *Pv*D_1_ from *Phaseolus vulgaris* (cv. Pérola) seeds. This protein was able to negatively affect different yeast cells and filamentous fungi [22], causing an increase in the endogenous production of ROS (reactive oxygen species) and NO (nitric oxide), plasma membrane permeabilization and the inhibition of medium acidification [23]. In the present study, we investigated the action of *Pv*D_1_ against the protozoan *Leishmania amazonensis*. We were also interested to understand the mechanism by which *Pv*D_1_ affects *L. amazonensis* promastigotes.

## EXPERIMENTAL

### Biological material

*P. vulgaris* L. seeds were supplied by the Empresa de Pesquisa Agropecuária do Estado do Rio de Janeiro (Pesagro), Campos dos Goytacazes, Rio de Janeiro, Brazil.

Promastigote-stage *L. amazonensis* (Josefa strain) were supplied by Laboratório de Biologia Tecidual, Centro de Ciências e Biotecnologia, Universidade Estadual do Norte Fluminense Darcy Ribeiro, Campos dos Goytacazes, Rio de Janeiro, Brazil. The protozoa were cultivated in 5 ml of Warren's medium [90% brain heart broth (Fluka) containing 10% (v/v) heat-inactivated FBS], enriched with 0.01% folic acid and 0.4% haemin at 28°C. The protozoa were transferred to new medium every 3 days.

### Purification of the *P. vulgaris* defensin *Pv*D_1_

The purification of the defensin from *P. vulgaris* (cv. Pérola) seeds was conducted as described by Games et al. [22].

### Gel electrophoresis

*Pv*D_1_ defensin purification was monitored by SDS/Tricine gel electrophoresis performed according to the method previously described by Schägger and Von Jagow [24].

### Cell proliferation assay

The effect of *Pv*D_1_ on the proliferation of *L. amazonensis* promastigotes was determined by incubating the parasites (1.5×10^6^ parasites/ml) with the *Pv*D_1_ defensin (300 and 600 μg/ml) for 48 h at 28°C in 200 μl microplate wells. The peptide was diluted in DMSO (1.5% final concentration) and Warren's medium (100 μl) and incubated with the parasites in Warren's medium (100 μl). The proliferation of the parasites was monitored at 24 and 48 h by cell counting in Neubauer chamber. The proliferation of *L. amazonensis* promastigote controls (without the addition of peptides) was also assessed. The experiments were performed in triplicate, and S.E.M. were calculated.

### Plasma membrane permeabilization assay

Membrane permeabilization of *L. amazonensis* promastigotes was assessed by measuring Sytox Green uptake as described previously, with some modifications, by Thevissen et al. [25]. Sytox Green is a dye that only penetrates cells when the plasma membrane is structurally compromised. Once inside the parasitic cytoplasm, it binds to nucleic acids, resulting in a fluorescent complex. The *L. amazonensis* promastigotes (1.5×10^6^ parasites/ml) were incubated with *Pv*D_1_ at a concentration of 300 μg/ml for 24 h at 28°C in 200 μl microplate wells. Aliquots (100 μl) of the parasite cell suspension were incubated with 0.2 μM Sytox Green in 1.5 ml microcentrifuge tubes for 30 min at 25°C with periodic agitation. After incubation, the parasites were fixed in 2% (w/v) paraformaldehyde. The cells were examined using a DIC (differential interference contrast) microscope (Axiophoto, Zeiss) equipped with a fluorescence filter set for the detection of fluorescein (excitation wavelengths, 450–490 nm; emission wavelength, 500 nm). Negative (no *Pv*D_1_ added) controls were also run to evaluate baseline membrane permeability.

### TEM (transmission electron microscopy)

For ultrastructural analyses, untreated or treated *L. amazonensis* promastigotes were washed with PBS at 37°C and fixed at room temperature in a solution containing 1% (w/v) glutaraldehyde, 4% (w/v) paraformaldehyde, 5 mM CaCl_2_ and 5% (w/v) sucrose in 0.1 M cacodylate buffer (pH 7.2). The cells were post-fixed for 1 h in a solution containing 2% OsO_4_, 0.8% potassium ferrocyanide and 5 mM CaCl_2_ in 0.1 M cacodylate buffer (pH 7.2), rinsed with 0.1 M cacodylate buffer (pH 7.2), dehydrated in acetone and embedded in PolyBed (Polysciences). Thin sections were stained with uranyl acetate and lead citrate and subsequently examined using a Zeiss 900 Transmission Electron Microscope at 80 kV acceleration.

### Intracellular localization of FITC-conjugated *Pv*D_1_

*Pv*D_1_ was conjugated to FITC (Sigma), according to the manufacturer's instructions. The *L. amazonensis* promastigotes (1.5×10^6^ parasites/ml) were incubated with *Pv*D_1_–FITC at a concentration of 150 μg/ml for 24 h at 28°C in 200 μl microplate wells. The cells were examined using a DIC microscope equipped with a fluorescence filter set for fluorescein detection (excitation wavelengths, 450–490 nm; emission wavelength, 500 nm).

## RESULTS

### Cell proliferation assay

The effect of *Pv*D_1_ on the proliferation of *L. amazonensis* promastigotes was evaluated. Figure 1 shows the inhibitory effects of *Pv*D_1_ (at concentrations of 300 and 600 μg/ml) towards the proliferation of parasites for periods of 24 h and 48 h. When compared with the control parasites grown in the absence of *Pv*D_1_, inhibitory rates of 70 and 89% were observed when testing *Pv*D_1_ at a concentration of 300 μg/ml at 24 h and 48 h respectively, whereas at a concentration of 600 μg/ml, inhibition rates of 87% and 96.5% were observed at 24 h and 48 h respectively.

**Figure 1:**
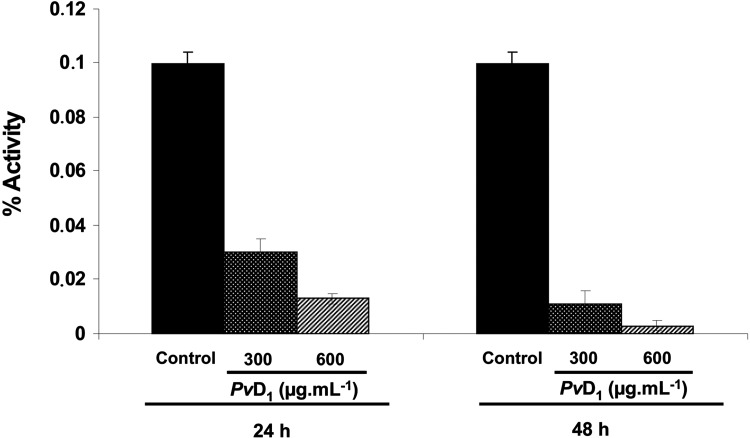
Inhibition of the proliferation of *L. amazonensis* promastigotes in the presence of *Pv*D_1_ Proliferation was observed for a 48 h period. The proliferation of *L. amazonensis* promastigote controls (without the addition of peptide) was also assessed. The experiments were performed in triplicate, and results are means±S.E.M.

### Plasma membrane permeabilization assay

In order to investigate further the negative effects of *Pv*D_1_ on *L. amazonensis* promastigote proliferation, we analysed whether cell membranes had been disrupted by the defensin. At 24 h of the proliferation inhibition assay, *L. amazonensis* promastigotes were treated with the fluorescent dye Sytox Green which only penetrates cells with structurally compromised plasma membranes. The observation of these cells by fluorescence microscopy showed that the *L. amazonensis* promastigotes were labelled by Sytox Green when treated with 300 μg/ml *Pv*D_1_ (Figure 2). In control cells, when parasites were grown in the absence of *Pv*D_1_, no fluorescence was observed.

**Figure 2:**
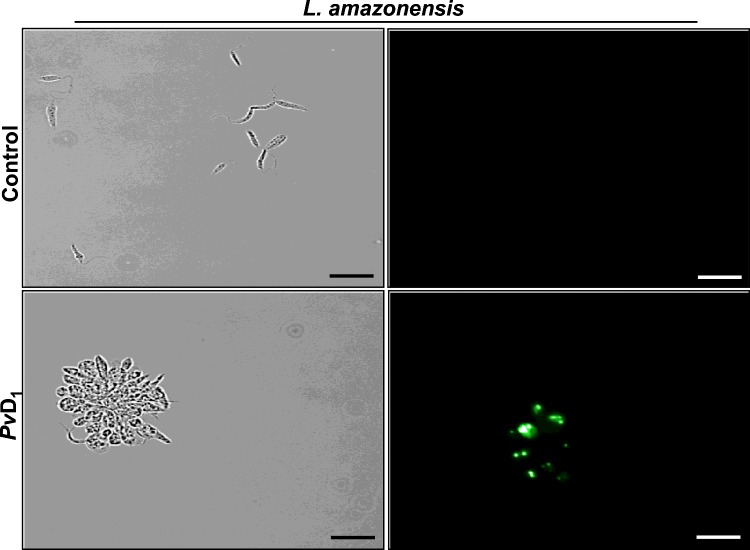
Plasma membrane permeabilization assay *L. amazonensis* promastigotes treated for 24 h with *Pv*D_1_ (300 μg/ml) and incubated with Sytox Green. The proliferation of *L. amazonensis* promastigote controls (without the addition of peptide) was also assessed. Scale bars, 20 μm.

### Ultrastructural analysis of *L. amazonensis* promastigotes treated with *Pv*D_1_

To confirm the effect of *Pv*D_1_ defensin on membrane permeabilization of *L. amazonensis* promastigotes, these cells were analysed by TEM. The ultrastructure analysis of promastigotes treated with 300 μg/ml *Pv*D_1_ revealed that this defensin was able to cause plasma membrane disruption followed by leakage of cytoplasmic material. It also caused cytoplasmic fragmentation and vacuolization. In contrast, control cells exhibited membrane integrity and normal intracellular organization (Figure 3).

**Figure 3:**
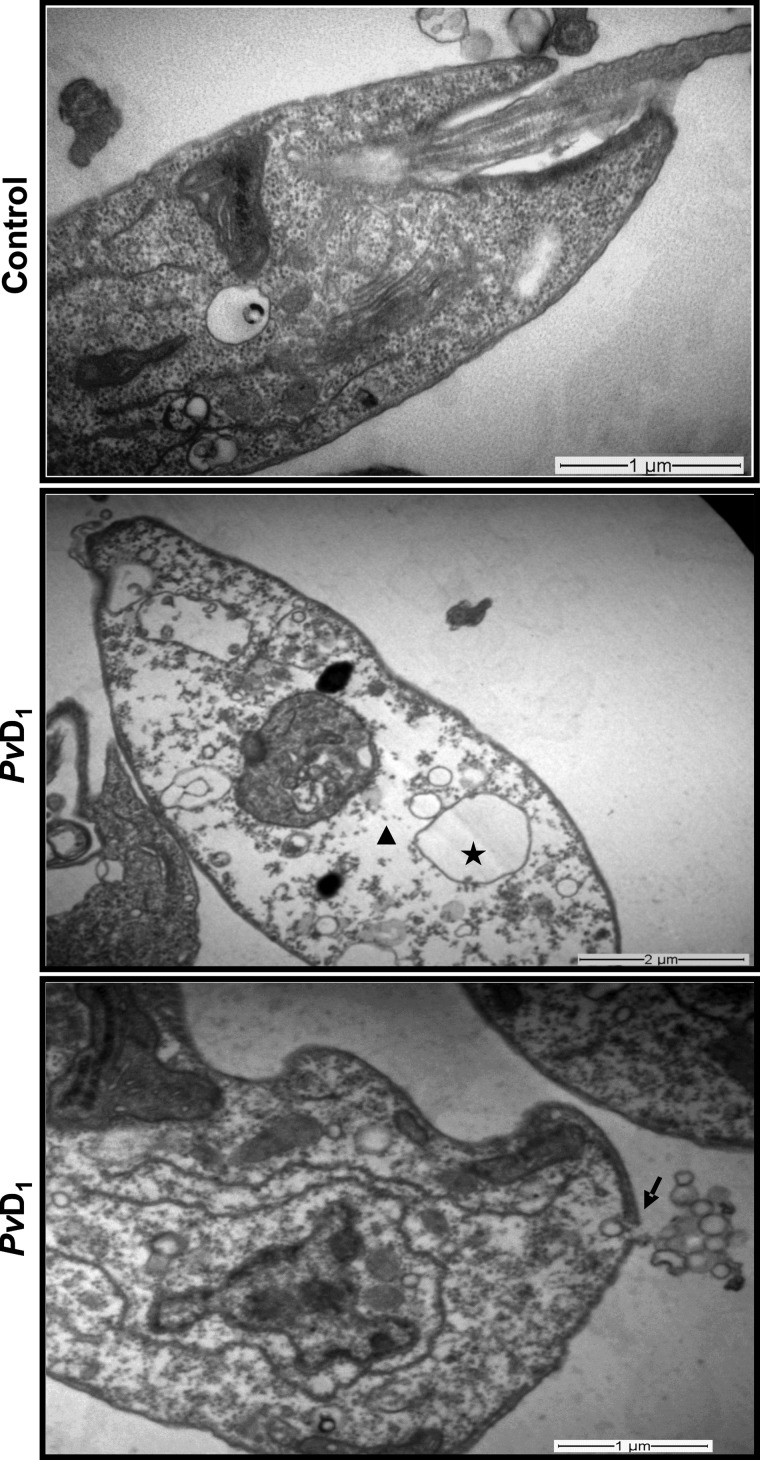
Ultrastructural analysis of *L. amazonensis* promastigotes treated with *Pv*D_1_ *L. amazonensis* promastigotes treated for 24 h with *Pv*D_1_ (300 μg/ml). The star indicates vacuolization of the cytoplasm; the triangle indicates fragmentation of the cytoplasm; the arrow indicates disruption in the plasma membrane of promastigotes leading to loss of cytoplasmic material.

### Intracellular localization of *Pv*D_1_

In order to investigate further whether the *Pv*D_1_ defensin could be internalized in *L. amazonensis* promastigotes, its ability to penetrate and accumulate inside the cell was evaluated by coupling the peptide with FITC. After the *L. amazonensis* promastigote proliferation inhibition assay using 150 μg/ml *Pv*D_1_–FITC, we observed the presence of *Pv*D_1_–FITC in intracellular spaces of these cells (Figure 4), suggesting a possible intracellular target of *Pv*D_1_ in *L. amazonensis* promastigotes. In control cells, no fluorescence was observed.

**Figure 4:**
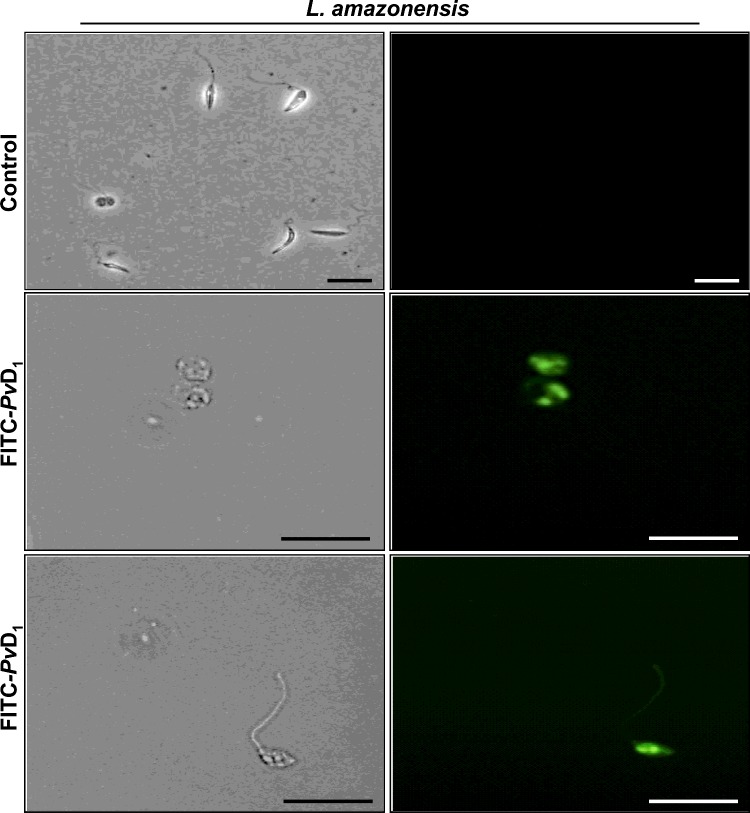
localization of FITC-conjugated *Pv*D_1_ *L. amazonensis* promastigotes were treated for 24 h with FITC*–Pv*D_1_ (150 μg/ml). The *L. amazonensis* promastigote control (without the addition of FITC–*Pv*D_1_) was also assessed. Scale bars, 20 μm.

## DISCUSSION

Plant defensins are capable of inhibiting the growth of a wide variety of filamentous fungi and yeast [5,6,23,22,26,27]. Despite the wide inhibitory activity that plant defensins have against different micro-organisms, there is a gap in our knowledge regarding the mechanism of action of plant defensins against protozoa. Only two studies to date show plant defensin activity against protozoan parasites of the genus *Leishmania* [28,29]. In the present study, we analysed the activity of a plant defensin, *Pv*D_1_, against *L. amazonensis* promastigotes and characterized some aspects of this plant defensin's action against this parasite.

We observed that at 300 μg/ml, *Pv*D_1_ was able to inhibit 70% of the protozoan proliferation, after 24 h of incubation (Figure 1). Berrocal-Lobo et al. [30] observed the activity of a PTH1 defensin from *Solanum tuberosum* and a thionin from *Triticum aestivum* against *L. donovani*; however, these authors did not test these peptides against *L. amazonensis*. More recently, Souza et al. [29] showed that the natural defensin from *Vigna unguiculata* seeds (*Vu*-Def), as well as its recombinant homologues (*Vu*-Defr), both at a concentration of 100 μg/ml, were also able to inhibit the proliferation of the parasite *L. amazonensis* by approximately 50% after 48 h incubation. Nonetheless, it is still unknown whether the plant defensins would affect other species of protozoa with similar strength.

After treatment of *L. amazonensis* promastigotes with *Pv*D_1_, we used the Sytox Green fluorescent dye which binds to nucleic acids when the cell plasma membranes are structurally compromised. We observed that *Pv*D_1_ was capable of causing damage to the plasma membrane (Figure 2), which was confirmed by the effects of *Pv*D_1_ on membrane permeabilization, seen using TEM. Using ultrastructural analysis, we showed that the cells of protozoa treated with the defensin exhibited a disruption of the promastigote plasma membrane leading to the loss of cytoplasmic material as well as cytoplasmic fragmentation and formation of multiple cytoplasmic vacuoles (Figure 3). Bera et al. [28] examined the effects of the AMP indolicidin (derived from bovine neutrophils) and two other peptides, 27RP and SPFK (derived from bovine seminal plasma), on *Leishmania donovani*. Interestingly, cells treated with these peptides exhibited extensive degeneration of intracellular organization and the formation of multiple cytoplasmic vacuoles without disruption of the plasma membrane. In contrast with the effect caused by AMPs, cells treated with amphotericin B exhibited no vacuolarization, although amphotericin B causes significant disruption of the plasma membrane. In the present study, we found both effects for *Pv*D_1_, which is the first reported plant AMP capable of causing cytoplasm fragmentation, formation of multiple cytoplasmic vacuoles and plasma membrane disruption of *L. amazonensis* promastigotes.

After this primary membrane-permeabilizing event, we analysed whether *Pv*D_1_ was able to internalize *L. amazonensis* promastigotes. For this, FITC-tagged *Pv*D_1_ was observed by fluorescence microscopy (Figure 4). The present study is the first to show that a plant defensin was able to enter *L. amazonensis* cells. On the basis of these data, we suggest the existence of a possible intracellular target for this defensin as a part of the mechanism responsible for leading to the protozoan's death.

In the present paper, we report the activity of a plant defensin against *L. amazonensis* and provide information on its mechanism of action. We demonstrate that, in addition to inhibiting the proliferation of *L. amazonensis* promastigotes, the *Pv*D_1_ defensin was able to cause cytoplasmic fragmentation, formation of multiple cytoplasmic vacuoles and membrane permeabilization in these cells. Furthermore, we show for the first time that *Pv*D_1_ defensin located within these cells may be acting on a possible intracellular target. These results open new perspectives regarding the antimicrobial mechanism of plant defensins as it suggests that the toxicity of these peptides may not be restricted to the plasma membrane.

## Abbreviations

AMP: antimicrobial peptide
DIC: differential interference contrast
